# Balance Training: Toward a Comprehensive Understanding and Application of the Overload Principle in Motor Skill Acquisition

**DOI:** 10.1002/ejsc.70130

**Published:** 2026-02-03

**Authors:** Tore Kristian Aune, Morten Andreas Aune, Vidar Estensen, Håvard Lorås

**Affiliations:** ^1^ Department of Sports Science Faculty of Education and Arts Nord University Levanger Norway; ^2^ Department of Teacher Education Faculty of Social and Educational Sciences NTNU Trondheim Norway

**Keywords:** exercise, motor control, performance, training

## Abstract

The effect of the overload principle in motor skill acquisition is unclear. Hence, the present study examined the effect of the overload principle in general, and in particular how the overload principle can be used to increase transferability to nontrained balance tasks with a high level of similarity at different levels of difficulty. A total of 24 participants were randomly assigned to two training groups: (1) low‐difficulty training group and (2) high‐difficulty training group. Both training groups completed five training sessions consisting of 25 trials over three weeks and only the level of difficulty of the balance board differed. Both the low and high‐difficulty training groups had a significant improvement in balance performance in the specific trained conditions, which supports the specificity principle. Most interestingly, only the high‐difficulty training group showed significant positive transfer to balance tasks with lower levels of difficulty. The low difficulty training group did not show significant positive transfer to balance tasks with higher levels of difficulty. These findings support the effect of the overload principle. In conclusion, the present findings demonstrate the superior effect of task‐specific balance training, and, most interestingly, the study revealed that training with overload at higher levels of difficulty enhances transferability to similar tasks performed at lower difficulty levels.

## Highlights

- The application and effect of the overload principle remain relatively under‐examined in the motor‐skill acquisition literature overall and particularly within the context of balance training.
- The present experiment explicitly aimed to answer the question about the effect of the overload principle on balance training and whether the overload principle can be used to increase transferability of balance to similar nontrained tasks of lower levels of difficulty.
- The experiment consisted of two groups: (1) high‐difficulty training group (HDTG) and (2) low‐difficulty training group (LDTG). Only the HDTG showed positive transfer to other balance tasks with lower levels of difficulty, whereas the LDTG did not induce positive transfer to balance tasks with higher levels of difficulty.

## 1 Introduction

Balance is a fundamental motor skill that underpins human movement and is essential for maintaining posture, stability, and coordination during various physical activities and involves the body's ability to maintain center of mass within the base of support. Balance denotes the capacity to effectively distribute body weight, thereby enabling stationary stance or movement without loss of equilibrium or facilitating recovery in the event of perturbation in activities of daily living, sporting endeavors, and other facets of daily existence (e.g., Shepard and Solomon 2000; Zech et al. 2010; Zemková 2014). Achieving proficient balance necessitates coordinated function across multiple neuroanatomical and physiological components, and impairments in any of these constituents can detrimentally impact an individual's balance capabilities (Massion et al. 2004).

Balance training is commonly recommended for various populations and is used for a variety of objectives. Studies have showed that balance training has the potential for enhancing athletic performance across various sports (Boccolini et al. 2013; Hrysomallis 2011; Zech et al. 2009, 2010), diminishing and preventing the risk of injuries, and expediting the rehabilitation processes (Herman et al. 2012; Hübscher et al. 2010; Krause et al. 2018; McGuine et al. 2000; McKeon and Hertel 2008; Sherrington et al. 2008). Additionally, it has been advocated as a preventive measure against falls within populations deemed susceptible (Gauchard et al. 1999; Mansfield et al. 2015).

However, to effectively evaluate and optimize the efficacy of balance training programs, it is crucial to deepen the understanding of the mechanisms underlying balance training effects. Current evidence demonstrates that balance training induces improvements solely in the specific tasks that are practiced and leads to limited generalizability and transfer across a broader range of balance tasks, including those not explicitly trained (Giboin et al. 2015, 2019; Henry 1968; Issurin 2013; Sayenko et al. 2012; Yaggie and Campbell 2006). A comprehensive understanding of which training principles most effectively promote performance improvement and transfer to related tasks, both in motor learning generally and balance training specifically, is essential for advancing evidence‐based practice and research. Such insights are essential for informing the development of appropriate study designs and testing protocols in this domain.

Several review studies have evaluated the effect of various balance training methodologies regarding whether balance training elicits predominantly task‐specific adaptations or more general applicable nonspecific adaptations with potential transferability to other balance tasks and other tasks that require elements of balance (McCrum et al. 2022; Kümmel et al. 2016; Lubetzky‐Vilnai and Kartin 2010; Tofthagen et al. 2012; Volery et al. 2017; Yavuzer et al. 2006).

Several studies have specifically aimed to address the issue of task‐specificity in balance training adaptations, and these investigations consistently report that, in healthy individuals, balance training enhances performance in the tasks that are directly practiced while exerting minimal or no effect on untrained tasks (e.g., Bakker et al. 2021; Donath et al. 2013, 2016; Giboin et al. 2015, 2019; Norman et al. 2012; Ringhof and Stein 2018; Rizzato et al. 2025; Wulf and Shea 2002). Consequently, it is essential to accurately identify the specific balance tasks requiring improvement and prioritize these tasks in both training protocols and assessment procedures. Accordingly, general balance tasks have limited utility in training interventions and testing, with task‐specific training and testing consistently demonstrating superior outcomes compared to more generalized approaches. The principle of specificity is fundamental to motor learning, with a central component being the task‐dependent nature of neural adaptations. Empirical research suggests that motor learning is facilitated through the repeated activation of distinct neural pathways, leading to task‐specific synaptic strengthening and functional reorganization within the central nervous system. This neuroplasticity process enhances the efficiency of neural circuits that correspond to the specific motor task being acquired (Carey et al. 2005; Chang 2014; Giboin et al. 2018; Rogge et al. 2018).

Another important training principle is the principle of progression and/or overload, and this principle remains relatively under‐examined in the motor‐skill acquisition literature overall and particularly within the context of balance training addressed in the present study. Both the concepts of progression and overload are established as important general training principles and applied in training for decades (Brearley and Bishop 2019; Hellebrandt 1958; Oxendine 1984; Walters 1956; Pearson et al. 2000). The overload principle is a well‐established concept in exercise physiology, emphasizing that to improve, a system must be challenged beyond its current capacity by imposing greater‐than‐normal demands on the body or motor system to induce physiological or neural adaptations (Carey et al. 2005; Muir et al. 2009). These aspects are in line with research on neuroplasticity, which have shown that the plasticity (adaptation and adjustment ability) of the neuromuscular system is substantial and increases because of specific training with progressive overload (Carey et al. 2005; Edelman 1987, 1992; Muir et al. 2009). In the context of motor skill acquisition, the concept of overload refers to increasing the difficulty and/or complexity of tasks that surpasses the existing motor skills an individual possesses to facilitate learning of new movement patterns and/or the refinement and optimization of existing skills (Walters 1956). Examples of how to overload from a behavioral perspective are to increase the level of difficulty (increase in level of motor or cognitive effort; e.g. speed, inclination) and/or complexity (increase in number of degrees of freedom; e.g. number of segments, perceptual, and attentional demands).

The overload principle is in line with the challenge point framework (CPF) trying to apply overload in a targeted manner strategically to find the optimal challenge point for the learner to promote skill acquisition (Guadagnoli and Lee 2004). The CPF partly emphasizes increasing task difficulty through the introduction of overload; however, when a learner struggles, it recommends reducing task difficulty to identify a more manageable challenge point that still facilitates learning. Despite the significance of the overload principle, the concept has received limited attention in motor skill acquisition research, particularly regarding how to effectively operationalize and apply the overload principle in practical settings for practitioners. Ignoring the overload principle can lead to reaching a plateau and stagnation in motor skill development, like what occurs in physical training, such as endurance and strength training (Burton and McCormack 2021; McCrum et al. 2022; Muir et al. 2009; Pearson et al. 2000).

The application of the overload principle in motor skill acquisition constitutes a pivotal inquiry and profoundly shapes potential recommendations for balance training interventions. Therefore, elucidating a more comprehensive understanding and application of the combination of the specificity and overload principle in motor skill acquisition in general, and in balance training in particular, will have the potential to guide the exploration of the underlying neurophysiological mechanisms governing adaptations induced by motor skill training. For example, if the effects of balance training are predominantly task‐specific, and the training effect increases with overload, it has important implications for the interpretation of the underlying mechanisms of adaptation. Task specificity at the behavioral level suggests that changes occur in a highly specific manner at the neurophysiological level (e.g., Black et al. 1990; Carey et al. 2005; Schubert et al. 2008). Such adaptations include strengthened neural representations of the practiced task within sensorimotor cortical areas, enhanced efficiency of task‐relevant motor unit recruitment, or refined integration of sensory information that is uniquely engaged during specific training with overload. These changes probably result from experience‐dependent plasticity, where repeated performance of a given motor task selectively reinforces the synaptic connections and neural pathways relevant to the specific task executed. (e.g., Boyd et al. 2010; Carey et al. 2005).

All training involves the concept of *transfer of learning* because of the need to transfer the learned capabilities from a situation to another, and in addition, how learning a skill can be facilitated (or impeded) by an already existing skill. In the motor learning literature, transfer refers to how practice under one set of task constraints influences performance under related but modified constraints, even when the task goal remains the same (Schmidt et al. 2018, Schmidt and Lee 2025a, 2025b; Magill and Anderson 2021). As described, both the specificity and overload principles are likely to have a positive transfer effect on motor skill acquisition in general and balance training in particular. Both the theory of *identical elements* (Thorndike 1903, 1913; Thorndike and Woodworth 1901) and the concept of *coordinative affinity* (Keller et al. 2023; Muller et al. 2000) provide theoretical frameworks for understanding the relationship between the training principles of specificity and overload in motor learning in general and in the transfer of learning in particular. Both approaches emphasize the critical role of similarity between training and competition contexts, not only with respect to environmental conditions but also regarding the coordinative structures of the skill at the behavioral level of analysis. Furthermore, these frameworks posit that such similarity ensures the induction of appropriately specific stimuli within the neuromuscular system, thereby facilitating effective adaptation and transfer.

The present experiment explicitly aims to answer the question about the effect of the overload principle on balance training and whether the overload principle can be used to increase transferability of balance to similar nontrained tasks of lower levels of difficulty. Based on these considerations, it hypothesized that practicing a balance task under conditions with heightened levels of difficulty (overload) would amplify transfer to an untrained but closely related balance task (high level of similarity and/or coordinative affinity), whereas practicing low‐difficulty conditions would not elicit such transfer.

## 2 Methods

### 2.1 Participants

A total of 24 volunteers (mean age 23.8, SD = 3.2 years; mean weight 80.8 kg, SD = 9.4 kg; height 176.9 cm, SD = 9 cm)., 11 females and 13 males, with no known neuromuscular problems, injuries, or diseases associated with balance impairment were recruited and gave informed written consent prior to participating in the study (see Table 1 for more details). The sample size was determined based on previous motor learning studies, employing similar experimental paradigms, where comparable group sizes have successfully identified significant training and transfer effects (Aune et al. 2017; Giboin et al. 2015) with moderate main effects at *d* = 0.50, *p* < 0.01, *β* = 0.90. The subjects had no specific balance training prior to the experiment, but had backgrounds from different sports (e.g., soccer, strength training, handball, gymnastics, and dancing). The subjects were divided into two training groups: low‐difficulty training group (LDTG) high‐difficulty training group (HDTG).

**TABLE 1:** Descriptive statistics (mean, SD) of the anthropometric for both low and high‐difficulty training groups.

Variable	LDTG (*n* = 12)	HDTG (*n* = 12)
Male/female (*n*)	7/5	6/6
Age (years)	23.92 (3.3)	23.75 (3.9)
Body weight (kg)	82.4 (7.7)	79.1 (7.1)
Height (cm)	183 (8.2)	175 (7.6)

Abbreviations: HDTG, high‐difficulty training group; LDTG, low‐difficulty training group.

All participants were right‐foot dominant, and footedness was established based on the following two assessments: (1) the subjects were asked to skip on one foot along a 5‐m line, and the foot used for skipping was defined as the preferred one, and (2) the subjects were pushed gently from behind, and the foot, the subject put forward to recreate balance, was defined as the preferred one (Schneiders et al. 2010). The study and experimental protocols were reviewed and approved by *Sikt* (Norwegian Directorate for Higher Education and Skills), ensuring compliance with relevant ethical and research guidelines (reference number 410578). Prior to participation, all participants provided written informed consent and completed both testing and training in accordance with the principles outlined in the Declaration of Helsinki.

### 2.2 Balance Learning Task

**FIGURE 1:**
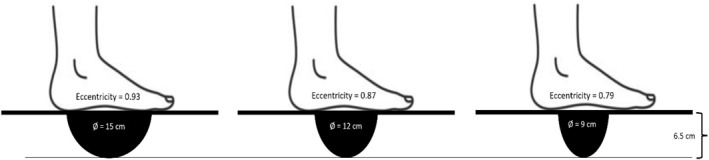
Illustration of the three levels of difficulty quantified by calculating levels of eccentricity of each wobble board.

**FIGURE 2:**
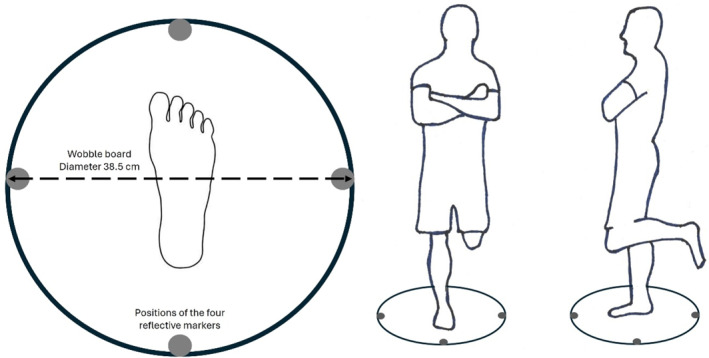
Illustration of the wobble board including positions of the four reflective markers (gray circles) and position of the subject standing on the wobble board.

The overall task was to stand unipedal on the preferred foot on a 40‐cm diameter wooden wobble board with a maximal 3D tilting angle of 20° in three levels of difficulty: (1) low difficulty, (2) medium difficulty, and (3) high difficulty. The subjects were instructed to keep the board as horizontally stable as possible during 20 s for each trial. The task was performed barefoot and with their arms crossed over their chest. In each trial, the subjects were instructed to position their preferred foot in the middle of the board while maintaining the balance on the nonpreferred foot positioned at the floor beside the board. The board was tilted with the edge touching the floor. Then, the subject was instructed to lift the nonpreferred foot off the ground and try to keep balancing the board horizontally and maintain equilibrium unipedal with the preferred foot. If the subjects lost control of the board and the edge of the platform touched the floor, or they started to use arms to keep balance, they had to repeat the starting procedure. Proper execution was controlled by the experimenter and corrected when necessary. See Figures 1 and 2 for more detailed information.

### 2.3 Apparatus

A standard “Select Profcare” (www.select‐sport.com) wooden wobble board was used consisting of a wooden platform with a rubber gripping surface, mounted on a spherical wooden block with a height of 5.5 cm. Together with the wooden platform that was 1 cm thick, the wobble board had a total height of 6.5 cm. To perform the task in the three different levels of difficulty (low, medium, and high), custom made versions of the spherical wooden blocks were used (see Figure 1). In all conditions, the wobble board had the same type of surface at the top of the platform, and the total height of the platform was the same in all conditions; but the spherical wooden blocks had different eccentricity that manipulated the level of difficulty between the three boards.

The eccentricity of the spherical wooden blocks was calculated using the following formula for eccentricity: ***e* = *c*/*a***. In the formula, ***c*** is the distance from the center to the minor axis, and ***a*** is the distance from the center to the major axis (the vertex). Eccentricity quantifies the deviation of an ellipse with reference to the perfect circle, and in this context, it can be used to describe the contact area between the spherical wooden block and the floor. An eccentricity value approaching 1 indicates a circular shape and, in this study, resulting in a greater contact surface with the ground. Conversely, an eccentricity value close to 0 signifies a lower contact surface with the ground. The custom‐made wobble boards utilized in this study exhibited the following eccentricity values: (1) low difficulty = 0.93, (2) medium difficulty = 0.87, and (3) high difficulty = 0.79 (See Figure 1).

The stability and movement of the wobble board was measured by four spherical reflective markers, 19 mm in diameter, attached perpendicularly along the perimeter of the wobble board (see Figure 2) and recorded with eight Oqus cameras via a motion‐tracking system (Qualysis, Sävedalen, Sweden, https://www.qualisys.com/). Kinematic data were recorded with Qualisys Track Manager 2.4 at a sampling rate of 400 Hz. The raw kinematic data were exported from the Qualisys Track Manager for further analysis by in‐house algorithms in MATLAB 24.1 (MathWorks, Natick, MA, USA).

### 2.4 Procedure

The experiment consisted of a pretest‐posttest design, separated by a training period. On the first day of the experiment, the subjects performed with three levels of difficulty of the balance task. For each condition, the subjects performed 5 trials of 20 s, separated by 20 s of rest. The first 2 trials in each condition served as familiarization trials and were not recorded, whereas the last 3 trials were used for performance assessment. Between each condition, the subject rested for 2 min. The order of conditions tested was counterbalanced across participants.

After the pretest, participants were allocated to two training groups using a matched‐pair assignment procedure based on their pretest balance performance. Balance performance was quantified as the ability to horizontally stabilize the balance board on the medium‐difficulty platform. This quasi‐randomization ensured that both groups exhibited comparable baseline balance skill levels at the onset of the training period: (1) low‐difficulty training group (LDTG) and (2) high‐difficulty training group (HDTG). The LDTG was selected to train the board with lowest difficulty (eccentricity = 0.93), whereas the HDTG was selected to train with the board with highest difficulty (eccentricity = 0.79). No board training was conducted with a medium level of difficulty. Five training sessions were completed over 3 weeks. Across the 3‐week training period, participants completed five training sessions, with an average interval of 3 days between each session, with a criterion of at least one day of rest between each session. Each training session consisted of 25 trials, divided into five blocks of five trials each. Each trial lasted 20 s with 20 s of rest between, and the subjects had 1 minute of rest between blocks to avoid fatigue. The total time of each training session was approximately 25 min. The participants were instructed to not conduct any other specific balance training while participating in the study. On the last day of the experiment, the subjects performed the posttest, which was like the procedure in the pretest.

### 2.5 Data Analysis

Raw kinematical data were initially prepared for further processing by means of a Butterworth 2nd order, low pass filter at 15 Hz with a zero phase (Robertson and Dowling 2003). To establish the horizontal stability of the balance board, designated as the dependent variable, the marker movement in Z‐position (vertical) was used for further analysis. First, a time series of the difference in position for distant markers were calculated (forward/backward and sideways tilt), in which zero indicates that the board is level with the floor (see Figures 1 and 2). Thereafter, the root mean square (Duarte and Freitas 2010) of the time series was calculated by the following formulas: $\surd (\sum \left({\Delta \;x}^{2}\right)/I\left(\Delta \;x\right)$ in which *Δ* x is the vertical movement of the wobble board, and thus constituted the performance measure of steadiness/tilt of the balance board.

### 2.6 Statistical Analysis

The average RMS of the three trials in each condition (for each subject) was used for further analysis. Normal distribution was inferred from Shapiro–Wilk tests, as well as inspection of Q‐Q plots and histograms. Thus, two (LDTG or HDTG group) × two (pretest or posttest) × three (low, medium, or high‐difficulty test) within‐subject repeated measures ANOVA was conducted on the RMS. In the rm‐ANOVA, partial eta squared (*η2p*) was applied as the indicator of the effect size and interpreted as small effect, 0.01; medium effect, 0.06; and large effect, 0.14 (Cohen 1988; Richardson 2011). Post hoc Bonferroni‐corrected pairwise comparisons were computed at the level of simple main effects, as well as for pre‐ and posttest differences for each test condition (low, medium, or high difficulty) within each training group (LDTG or HDTG group). For these post hoc tests, Cohen's *d* was applied as a measure of the effect size (Lakens 2013), in which 0.2, 0.5, and 0.8 were interpreted as small, moderate, and large, respectively (Cohen 1988). The statistical calculations and analyses were performed in IBM SPSS Statistics (version 29.0, SPSS Inc., Chicago, IL, United States), with alpha an level of *p* ≤ 0.05 as the criterion for statistical significance.

## 3 Results

**FIGURE 3:**
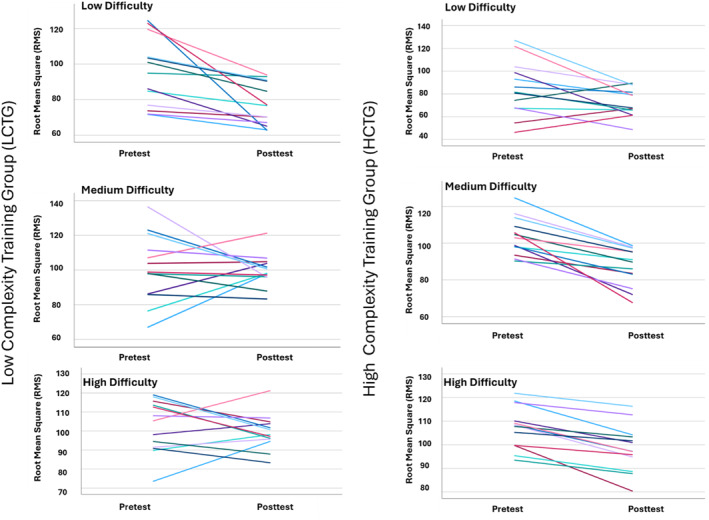
Overview of individual learning and transfer effects for both the low and high‐difficulty training groups between pretest and posttest.

An overview of results across groups and conditions is illustrated in Table 2 and Figure 3. A repeated measure (rm) ANOVA indicated no significant group (LDTG vs. HDTG) × time (pretest vs. posttest) × condition (low, medium, or high difficulty) interaction effect on RMS [*F* (1,23) = 3.23, *p* = 0.07, *η2p* = 0.21]. Further, rm‐ANOVA indicated a significant main effect of time (pretest vs. posttest: *F* (1, 23) = 28.65, *p* > 0.001, *η2p* = 0.72) and a significant main effect of test (low, medium, or high difficulty: *F* (1, 23) = 28.37, *p* > 0.001, *η2p* = 0.70). Bonferroni‐corrected pairwise post hoc comparisons demonstrated significant RMS reduction from pretest to posttest for low difficulty [*Δ* = −15.07, 95% CI [−22.51, −7.64], SE = 3.41, *p* < 0.001, *d* = 0.90] medium difficulty [*Δ* = −8.98, 95% CI [−15.42, −2.54] SE = 2.96, *p* = 0.010, *d* = 0.62], and high difficulty [*Δ* = −6.42, 95% CI [−9.63, −3.21], SE = 1.47, *p* < 0.001, *d* = 0.89] assessments. There were no significant between‐subject group effects on the RMS from pretests (*M* = 0.585, *95%* CI [−9.92, 11.61], *F* (1, 12) = 0.029, *p* = 0.867, *η2p* = 0.002).

**TABLE 2:** Descriptive statistics (mean, SD) for both low and high‐difficulty training groups at pretest and posttest.

Variable	*Pretests (rms)*	*Posttest (rms)*
LDTG (*n* = 13)		
High	102.35 (13.83)	98.36 (9.08)
Medium	101.00(19.47)	99.63 (9.19)
Low	95.07 (19.34)	77.29 (11.91)*
HDTG (*n* = 13)		
High	107.35 (8.72)	98.50 (9.82)*
Medium	103.66 (10.24)	87.07 (10.37)*
Low	84.89 (24.03)	72.51 (12.55)*

Abbreviations: HDTG, high‐difficulty training group; LDTG, low‐difficulty training group, RMS, root mean square.

^*^ Indicates significant differences between pre‐ and posttest (*p* < 0.05).

Further analysis of the HDTG that practiced on the most difficult wobble board (see Figure 1) indicated that for the low‐difficulty test, HDTG participants showed a significant improvement from pretest to posttest (*M* = 12.37, SE = 5.49), *p* = 0.044, 95% CI [0.41, 24.34], with a Cohen's *d* of 0.92, indicating a large effect. For the medium‐difficulty wobble board, performance also significantly improved from pretest to posttest (*M* = 16.59, SE = 2.59), *p* < 0.001, *95%* CI [10.94, 22.23], with a very large effect size, *d* = 1.39. Similarly, for the practiced high‐difficulty wobble board, HDTG participants improved significantly (*M* = 8.84, SE = 1.36), *p* < 0.001, 95% CI [5.88, 11.81], with *d* = 1.46. In contrast, the LDTG group that practiced the low‐difficulty wobble board (Figure 1) showed a significant improvement only at the practiced low‐difficulty test, with pretest to posttest improvement (*M* = 17.78, SE = 4.93), *p* = 0.004, 95% CI [3.08, 28.51], corresponding to a large effect size of *d* = 1.45. However, no significant improvements for LDTG participants were observed at the medium‐difficulty wobble board (*M* = 1.38, SE = 5.43), *p* = 0.805 or the high‐difficulty wobble board (*M* = 3.99, SE = 3.14), *p* = 0.227.

## 4 Discussion

The present experiment is the first study by the author's knowledge that explicitly aimed to answer the question about the effect of the overload principle in balance training, and it was hypothesized that practicing a balance task under conditions with heightened levels of difficulty (overload) would amplify transfer to an untrained but closely related balance task, whereas practicing low‐difficulty conditions would not elicit such transfer.

Both the low and high‐difficulty training groups had a significant improvement in balance performance in their specific trained condition, which supports earlier research describing the specificity of motor learning in general, and in balance tasks in particular (Giboin et al. 2015, 2019; Henry 1968; Issurin 2013; Sayenko et al. 2012; Yaggie and Campbell 2006). Most interestingly, only the high‐difficulty training group showed a significant positive transfer to other balance tasks, whereas the low‐difficulty training group did not induce a significant positive transfer to other conditions. More specifically, the high‐difficulty training group showed positive transfer to balance tasks with lower levels of difficulty, respectively the task of medium similarity and medium difficulty and the task with low similarity and low difficulty, (see Table 2 and Figure 3). However, the low‐difficulty training group did not induce a significant positive transfer to balance tasks with higher levels of difficulty, respectively the task of medium similarity and medium difficulty and the task with low similarity and high difficulty (see Table 2 and Figure 3). Even though maintaining the balance on all three wobble boards was observed as demanding, training with the high‐difficulty board led to transfer across tasks, whereas training with the low‐difficulty board improved performance only in the practiced condition. These findings support the hypothesized positive effect of training with overload for enhancing transfer to an untrained but closely related balance tasks (high level of similarity and/or coordinative affinity), and that practicing with low‐difficulty conditions does not elicit such transfer effects.

A comprehensive understanding of the mechanisms underlying balance as a motor coordination skill is crucial, particularly in relation to evaluating and optimizing the efficacy of balance training programs. An important question remains whether balance training yields improvements exclusively in the practiced tasks or also produces generalized enhancements across untrained balance tasks, and the issue holds significant relevance for both practitioners and researchers. The present findings demonstrate that task‐specific training is both highly effective and superior to generalized training. These findings align with previous experimental studies and meta‐analyses that have examined the effects of various balance training methodologies, consistently reporting that in healthy individuals, balance training improves performance primarily in the specific tasks that are directly practiced (e.g., Bakker et al. 2021; Giboin et al. 2015; Ringhof and Stein 2018). However, the transfer of balance skills appears to occur only when training involves an overload condition, such as increased task difficulty in the current study.

The main purpose of the study was to investigate the application of the overload principle to motor skill acquisition in general (Brearley and Bishop 2019; Oxendine 1984; Walters 1956) and, specifically, toward balance training. The present results indicate that, to facilitate motor skill transfer, individuals must be sufficiently challenged by exceeding the current level of motor skill an individual possesses. Specifically, overload induced through training at high levels of difficulty is necessary to achieve transferability to even similar tasks of lower difficulty levels. These observations at the behavioral level of analysis in the present study are in line with research on neuroplasticity, which has demonstrated that plasticity (adaptation and adjustment ability) of the neuromuscular system is substantial and increased because of specific training involving progressive overload (Carey et al. 2005; Edelman 1987, 1992). In addition, it is suggested that neglecting the principle of overload may result in a plateau and stagnation in motor skill development, akin to the patterns observed in physical training domains such as endurance and strength development (McCrum et al. 2022; Friedmann‐Bette et al. 2010; Shirai et al. 2021).

These findings can also be interpreted through the CPF (Guadagnoli and Lee 2004), where high‐difficulty practice likely placed participants closer to their optimal challenge point, supporting learning beyond the practiced task. In contrast, low‐difficulty practice remained below this threshold and limited transferable effects, as more difficult tasks exceeded participants' coordinative capacity. This interpretation is consistent with Thorndike's theory of identical elements (1901, 1903), since the low‐difficulty task lacked critical postural and coordinative demands such as rapid error correction, multidirectional stability control, and heightened attentional engagement. Even though the trained and untrained balance tasks were structurally similar, the absence of these elements appears to have limited transfer.

Although the CPF explains why higher task difficulty is necessary to facilitate learning, it does not fully account for how the motor output was expressed in achieving improved stability. In the current study, the reduction in RMS was used as an indicator of motor output and performance improvement in balance stability. The present study did not directly assess the coordination mechanisms underlying balance performance. Nonetheless, the reduction in RMS suggests an improvement in postural stability. Training at higher difficulty levels may have exposed participants to more demanding sensorimotor conditions (Schedler et al. 2020), which could account for the observed performance enhancements. This interpretation is consistent with previous work demonstrating that training on unstable surfaces can improve proprioceptive functioning, sensorimotor integration, and task‐specific neuromuscular adaptations (Taube et al. 2007). Increased task difficulty may therefore impose greater sensory and neuromuscular demands, thereby contributing to the improvements observed in the current study. However, the present data do not allow for identification of the specific neural mechanisms or coordinative changes involved, which should be addressed in future research.

### 4.1 Practical Implications

The present findings are consistent with previous research considering balance training as highly task‐specific, and the fact that it is a need to reconsider the use of “balance” and “balance training” as general terminology, as it implies that balance is a general ability with broad transferability across tasks. Moreover, meta‐analyses that aggregate various forms of “balance training” should be interpreted cautiously as the variability in training methods and objectives complicates direct comparisons. In terms of more practical applications, the findings of this study suggest that training programs should first identify the specific balance tasks and their identical elements requiring improvement and then develop a training regimen specifically tailored to those tasks. Furthermore, researchers should reevaluate the use of generic balance task assessments in studies related to sport science, rehabilitation, and aging populations to more accurately assess the efficacy of, for example, different training regimens.

Advancing a comprehensive understanding and application of the specificity and overload principles in motor skill acquisition research, such as in balance training, holds considerable potential for informing the investigation of neurophysiological mechanisms underlying adaptations induced by motor skill training more broadly. For example, if the effects of balance training are predominantly task‐specific, future research could explore corresponding neurophysiological adaptations that reflect the specificity of the training stimuli.

Among coaches and therapists, balance training is widely recommended and applied to achieve a variety of objectives. The present findings suggest that generalized balance tasks offer limited utility in both training interventions and assessments, and in contrast, task‐specific training and testing consistently demonstrate superior outcomes compared to more generalized approaches. For practitioners, these results underscore the importance of accurately identifying the specific balance tasks requiring improvement and prioritizing them in both training protocols and assessment procedures. Most interestingly, the present study highlights the crucial importance of incorporating overload and individually tailored progression to optimize training outcomes. For instance, in injury and fall prevention programs, selecting training tasks that closely replicate those posing the greatest risk of falls or injuries, and practicing them in controlled environments, appear to be particularly beneficial. Additionally, given the task‐specific nature of balance training effects, the use of generic balance tests may not be suitable for evaluating adaptations across tasks with varying levels of difficulty and complexity.

### 4.2 Limitations of Study and Future Studies

Despite the present results that seem to be quite unequivocal, there are some limitations that warrant some further examination. The duration of the study was not very long, and thus it could be possible that the training duration was not long enough to develop skills that could be transferred to other balance tasks that are of higher difficulty for the LDTG. The training period in this study was relatively short; however, this is justifiable in motor skill acquisition research as initial learning and adaptation typically occur rapidly, with participants developing fundamental task‐specific neural and motor patterns early in the learning process (Krakauer et al. 2019). The early phases of motor learning are marked by pronounced improvements, rendering short‐term interventions effective for investigating the basic principles of motor learning and skill transfer.

Furthermore, one might argue that the three levels of balance tasks examined all used one‐legged stance, potentially limiting the generalizability of the results, for example, to two‐legged stance and other activities in daily life and sport. Although this possibility cannot be totally excluded, it is unlikely that there should be a higher level of transferability from the present tested tasks with relatively high level of similarity to other tasks with less levels of similarity according to Thorndike's *identical elements theory* (1901; 1903). For future studies aiming to investigate the application of the specificity and overload principles in balance training and motor learning in general, it would be valuable to examine a range of balance tasks and motor skills across varying levels of specificity and overload. This approach could provide deeper insights into how the overload principles influence skill acquisition and skill transfer across various tasks with different difficulties and complexities.

Large sample sizes are often desirable for statistical power and generalizability, and in the present study, it is necessary to recognize the limitation of the relatively modest sample size for generalizability. Accordingly, although acknowledging the modest sample size, it is justified by both practical constraints and methodological rigor, consistent with established practices in the motor learning domain. The experiment could also have included a control group to test the presence or absence of the training effects, but because of a modest sample size, it was decided to divide the subjects into two training groups and test the relative effectiveness between them.

## 5 Conclusion

In conclusion, the present findings demonstrate the superior effect of task‐specific balance training, and most interestingly, the study revealed that training with overload at higher levels of difficulty enhances transferability to similar tasks performed at lower difficulty levels. In contrast, no significant transfer effects were observed from training at lower difficulty levels to similar tasks at higher difficulty levels. Based on these findings, it is recommended that balance training programs prioritize highly task‐specific exercises. When incorporating training variability, where the specific balance task is not directly trained, it is crucial to implement overload through tasks that share high levels of similarity and contain identical elements to the target task.

## Funding

The authors have nothing to report.

## Conflicts of Interest

The authors declare no conflicts of interest.
